# Stylalgia Revisited: Clinical Profile and Management

**Published:** 2018-11

**Authors:** Junaid-Nasim Malik, Seema Monga, Arun-Parkash Sharma, Nighat Nabi, Khaja Naseeruddin

**Affiliations:** 1 *Department of Otorhinolaryngology and Head and Neck Surgery, Hamdard Institute of Medical Sciences and Research and HAHC Hospital, Jamia Hamdard university, Hamdard Nagar, Delhi-110062, India.*

**Keywords:** Chronic throat pain, Eagle’s syndrome, Stylalgia, Tonsillo-styloidectomy, Visual Analog Scale, Pregabalin

## Abstract

**Introduction:**

Eagle’s syndrome is a constellation of signs secondary to an elongated styloid process or due to mineralization of the stylohyoid or stylomandibular ligament or the posterior belly of the digastric muscle. The syndrome includes symptoms ranging from stylalgia (i.e. pain in the tonsillar fossa, pharyngeal or hyoid region) to foreign-body sensation in the throat, cervicofacial pain, otalgia, or even increased salivation or giddiness.

**Materials and Methods:**

We describe a clinical study of 12 patients with Eagle’s syndrome, along with their clinical profile and the treatment offered. Patients were diagnosed based on history and clinical examination, as well as the Xylocaine 2% tonsillar fossa injection test. A visual analog scale (VAS) was used for comparison of pain before and up to 3 months after treatment. Radiology (orthopantomogram or three-dimensional computed tomography) was used for further exploration. Nine patients underwent tonsillo-styloidectomy surgery and three underwent medical treatment with pregabalin (75 mg/day).

**Results:**

The majority of surgically-managed cases (88%) achieved a definitive benefit by tonsillo-styloidectomy surgery, whereas all medically managed cases achieved only short-term pain relief.

**Conclusions:**

Besides the common throat diseases, the symptoms associated with Eagle’s syndrome may be similar to those due to cervicofacial neuralgias, dental, or temporo-mandibular joint diseases. Diagnosis is primarily based on symptomatology, physical examination and radiographic investigations, and should not be missed. Treatment by tonsillo-styloidectomy produces satisfactory results in stylalgia.

## Introduction

The styloid process is a cylindrical, spikey bony-cartilaginous structure arising from the inferior part of the petrous temporal bone. The normal length of the styloid process is 20–30 mm. Eagle’s syndrome is described as the symptomatic lengthening of the styloid process or mineralization (ossification or calcification) of the stylohyoid ligament complex. Various sources suggest the possibility that Eagle’s syndrome was identified as early as the 17^th^ century. However, the syndrome was named after Watt W. Eagle who described stylalgia in 1937 (1). The syndrome is also known as Long Styloid Process Syndrome or Styloid Process Neuralgia. The main symptoms of stylalgia are due to the anomalous length of the styloid process or the mineralization of the styloid ligament, or because of calcification of the digastric muscle. Patients may present with pharyngeal pain, and an irritating sensation in the throat and ear ache. There may also be cervicofacial pain radiation, usually a dull aching type. Head rotation as well as yawning or lingual movements may be painful.

It seems that diagnosis of the condition is liable to be missed because the number of cases in the population remain underestimated. This is due to the varied and non-specific characteristics of symptoms and the patient’s search for treatment within various specialties such as otolaryngology, neurology, dentistry, and psychiatry. In this study, we intend to describe various clinical and radiological profiles of patients with Eagle’s syndrome and evaluate the results after treatment.

## Materials and Methods

This was an institution-based study in which all patients diagnosed with Eagle's syndrome in our hospital during the 3-year duration from January 2013 to December 2015 were included. The follow-up period of these patients was a minimum of 3 months after treatment. 

The diagnosis of Eagle’s syndrome was made by a history of a chief complaint of intractable throat pain, either dull aching or a pricking sensation in the tonsillar region, which often radiated to the side of the neck, along with a tender feeling on palpation of the prominent styloid process in the tonsillar fossa. Coexistent gastro-laryngopharyngeal reflux (GLPR)/ gastroesophageal reflux disease (GERD) was treated to rule it out as the cause of chronic throat pain.

**Fig1:**
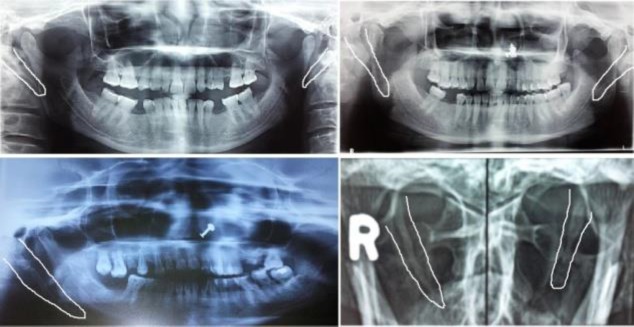
Multiple X-ray views showing elongated styloid process

Pain assessment was performed using a visual analog scale (VAS), a numerical-pictograph objective tool often employed for the measurement of pain severity. Patients were asked to indicate the severity of the pain felt according to a scale ranging from **0** (meaning no pain) to 10 (meaning severe intolerable pain); all patients had a VAS score between 7 and 9 at presentation. The lengthened styloid process was confirmed on radiology by a mouth-open Towne’s view X-ray or an orthopantomogram (OPG) (Fig.1).

**Fig 2:**
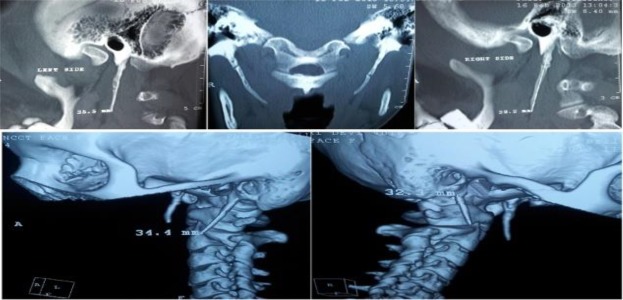
Various CT scan sections and with 3D reconstruction showing bilaterally elongated styloid processes

Additionally, computed tomography (CT) scans with three-dimensional (3D) recons- truction were performed (Fig.2).

A diagnosis of Eagle’s syndrome was further confirmed by the Xylocaine injection test, in which 2 ml of 2% Xylocaine was infiltrated into the tonsillar bed on the affected side with the palpable styloid process in a method similar to the one advocated by Singhania et al. (2). The patient was then asked to assess the pain level on the VAS again 10 minutes after the Xylocaine injection, and this was compared with the pre-injection pain level. Relief in pain intensity by more than one-third of the original pain levels after injection was taken as confirmation of stylalgia.

**Fig 3:**
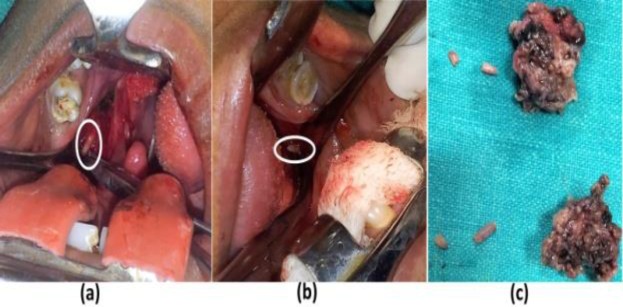
Intra-operative picture showing the elongated styloid processes during styloidectomy operation

Nine out of the 12 cases were treated surgically by intraoral styloidectomy (Fig.3).

These patients were followed up 1 week after discharge for a routine checkup and to rule out any developing complications. All patients were routinely followed up until 3 months, and at every visit the level of pain on the VAS was noted. The remaining three patients could not be operated on due to patient factors (elderly age [one patient], unfit for general anesthesia [one patient], and choice of non-surgical treatment [one patient]). These patients were managed conservatively with medical treatment (pregabalin, 75 mg/day) and showed pain relief by more than two-thirds on the VAS scale. At 3 months follow-up, a decrease in pain severity by more than two-thirds of the pre-operative score was considered successful treatment.

## Results

Out of a total of 12 patients, nine were female and three were male (female:male ratio, 3:1). The mean age of patients was 46 years, with the youngest patient presenting at 28 years and oldest at 80 years of age (Table.1).

**Table 1:** Clinical presentation of cases

**Age (years)**	**Male**	**Female**
21–30	0	1
31–40	2	3
41–50	1	4
51–60	0	0
>60	0	1
Symptoms	No. (%) cases	
Chronic throat pain	10 (83.3%)	
Odynophagia	8 (66.6%)	
Foreign-body sensation	4 (33.3%)	
Cervicofacial pain radiation	4 (33.3%)	
Referred Otalgia	2 (16.6%)	
Stylocarotid symptoms	None	

The duration of symptoms was less than 1 year in six cases, 1–3 years in four cases, and more than 3 years in two cases. Most (10/12) of the symptomatic cases were unilateral, while two were bilateral. Bilateral radiological elongation was seen in nine cases and unilateral elongation was seen in three out of the 12 cases. Radiologically, the longest styloid measured was 4.6 cm in length, while the shortest was 2.8 cm. 

Medial angulation in reference to a theoretical vertical midline on OPG or CT scan was observed in seven patients and anterior elongation was observed in three patients (Fig 2). Ten patients presented with chronic throat pain; eight had aggravation on swallowing; four had foreign-body sensation; six had referred pain, of which four had pain referred to the same side of the neck and two had referred earache. None of the patients presented with stylocarotid syndrome symptoms such as dizziness, vascular compression or headache (Table.1). 

Coexistent GERD/GLPR was seen in seven patients. During the 3 months of follow-up, four patients had complete pain relief, three by styloidectomy and one patient by pregabalin (VAS score,1). Seven patients achieved satisfactory (i.e. more than two-thirds) pain reduction, five patients by styloidectomy and two by medication (VAS score, 2–4). One patient treated by styloidectomy had no pain relief and continued with a VAS score of 8. 

After the end of 3 months follow-up, two out of a total of three patients treated medically had a recurrence of pain symptoms (VAS, 6–7) on discontinuing the treatment. The results were collected, tabulated, and analyzed as shown in (Table.2).

**Table 2:** Post-treatment outcomes

**Follow-up**	**No. cases treated surgically**	**No. cases treated medically**
Symptom-free (cured) at 2 weeks	4/9	2/3
Symptom-free (cured) at 1 month	7/9	3/3
Symptom-free (cured) at 2 months	8/9	3/3
Symptom-free (cured) at 3 months	8/9	3/3

## Discussion

Eagle first defined stylalgia as associated with a lengthened styloid process or due to mineralization of the stylohyoid ligament complex (1). It was later reported in various studies that patients with a long styloid process and those with misdirected angulation presented with maximum symptoms (3,4).

Eagle assessed the incidence of an elongated styloid process in the overall population to be 4%, of whom only 4% were symptomatic (5). Similarly, Bozkir et al. reported a frequency of 4%, while Kaufman et al. estimated elongated styloid process in 28% of patients in their clinical studies (6,7). However, radiological studies give different results. Correl et al. reported the incidence to be 18.2% by examining radiographs (1,711 panoramic views), 93% of which revealed bilateral elongation. However, only a minority of patients (eight out of 1,771) presented with symptoms of stylalgia, which were mostly unilateral (8). Similarly, in 2005, Rizzati-Barbosa et al. also studied a total of 2,252 panoramic radiographs and found a frequency of 20% (9). In 1948, Eagle stated that the styloid process was approximately 2.5 cm in length and that any styloid more than 2.5 cm long should be considered as elongated; by this definition, elongated styloid was observed in 4% of cases examined (1). In contrast, Kaufman et al. reported the average length of the styloid process as 2–3 cm in accordance with various other studies (7). If the styloid process exceeds 3 cm on X-ray, it is considered to be elongated (10), and reviews of the literature and radiological studies support that the length of the styloid process should not be greater than 25 mm and the upper limit should be taken as 3 cm in general (11,12). In our study the average length of the right styloid process was 3.8 cm, and 3.5 cm on the left side.

Although styloid process elongation is seen bilaterally in most cases, patients are usually symptomatic unilaterally for stylalgia despite the presence of bilateral elongation (13,14). The condition is generally noted as being more common, especially symptomatically, in females than in males (4,10,11,15). Styloid process elongation commonly occurs above the age of 30 years (16), and some studies suggest it to be more frequent among the elderly due to calcification of the ligaments and processes, possibly due to reactive hyperplasia/metaplasia or anatomical variance (17). In our study, the male-to-female ratio was 1:3 and the mean age of presentation was 46 years. An elongated or misdirected styloid process or a calcified stylohyoid ligament can point into the tonsillar fossa and impinge upon vital structures in the neck and pharynx, leading to various neurological or vascular symptoms (1). Chronic throat pain and aggravation of symptoms on swallowing were the two main symptoms in the present study. This is in accordance with various previous studies (4,18,19). Although there can be a wide spectrum of symptoms, the most common is chronic throat pain. Eagle described two types of syndrome complexes (1). The first is the classical type, which presents as throat pain, referred otalgia, and foreign-body sensation in the throat. Eagle associated these symptoms with scar tissue formation around the tip of the styloid process soon after tonsillectomy. The second type, stylocarotid or carotid artery type, presents as carotidynia, headaches, facial pain, and dizziness. This vascular form of the syndrome is not related to tonsillectomy and is attributed to impingement of the carotid artery and its sympathetic plexus, extracranially by the styloid process, with aggravation of symptoms upon head turning.

The syndrome is diagnosed clinically by digital palpation of the tonsillar fossa whereby a sharp pointed hard structure is felt, which on pressing aggravates the pain. Furthermore, stylalgia can be confirmed clinically by the Xylocaine injection test, in which 2% Xylocaine is injected into the tonsillar bed, after which the patient has a significant decrease in pain intensity (2). Radiologically, the condition is established by a panoramic radiograph (OPG) or Towne’s or AP skull view. To measure the actual length of the styloid process and analyze its course and adjacent anatomical relations, a CT scan with 3D reconstruction can be done (12), although its additional cost limits it widespread use. In our study, 3D CT was done in nine out of 12 patients. In the remaining three patients, this was not possible due to financial constraints.

Depending on the intensity of pain and odynophagia, treatment may vary from a medical to a surgical line of management. For surgical excision, two approaches have been used; the extraoral and the intraoral approach, both of which have various modifications and advantages. The extraoral approach is preferred by some authors mainly because of improved visualization and a lower chance of deep neck space infection. The intraoral approach is usually employed by otolaryngologists who are familiar with the technique of tonsillo-styloidectomy. In our study, all eight surgically treated patients were operated on by the intraoral procedure of tonsillo-styloidectomy. Fortunately, none of the operated cases developed any complications owing to meticulous surgical technique and good post-operative care. In 88% of patients, tonsillo-styloidectomy provided excellent relief at 4 weeks postoperatively. These observations are comparable with other studies. For example, Singh et al. reported a 95% success rate, and 96% success was observed by Yavuz et al. Similarly, Yadav et al. (20) reported that of 40 patients operated on, 31 (77.5%) became symptom-free, five (12.5%) had considerable symptomatic improvement, while four (10%) had no relief (4,19,20).Non-surgical treatment includes reassurance, non-steroidal anti-inflammatory drugs (NSAIDS), gabapentin, pregabalin, carbamazepine, tianeptine, amitriptyline, physiotherapy, steroid injections, and injections of long-acting local anesthetics and/or stellate ganglion block (12,17). In our study, non-surgical management was by pregabalin 75 mg/day in three patients. Although all three cases responded to medical management and one patient became pain free (cured), two cases had a reappearance of symptoms on stopping the medication. Pregabalin was effective in the management of stylalgia in our study, but only until continuous medication.

Differential diagnoses include various headaches, facial pain and neuralgias, neck pain, ear, nose, and throat diseases, psychosomatic disorders and inflammatory and neoplastic disorders in the head and neck region which need to be ruled out before concluding a diagnosis of Eagle’s syndrome in order to deliver the appropriate treatment (11,21).

## Conclusion

Eagle’s syndrome is an entity in which the symptoms are related to an elongated styloid process. Diagnosis mainly relies on a history and clinical examination, aided by radiology; however, not all elongated styloid processes seen radiologically cause stylalgia. We advocate styloidectomy as an estimable modality of the treatment in stylalgia, but proper selection of patients for surgery is important for proven cases of chronic throat pain due to Eagle’s syndrome so that maximum relief of symptoms is achieved.
